# PTH/SDF-1α cotherapy induces CD90+CD34− stromal cells migration and promotes tissue regeneration in a rat periodontal defect model

**DOI:** 10.1038/srep30403

**Published:** 2016-08-02

**Authors:** Fang Wang, Lingqian Du, Shaohua Ge

**Affiliations:** 1 Shandong Provincial Key Laboratory of Oral Tissue Regeneration, School of Stomatology, Shandong University, Jinan, China; 2 Department of Periodontology, School of Stomatology, Shandong University, Jinan, China

## Abstract

Stromal cell-derived factor-1α (SDF-1α) is a key stem cell homing factor that is crucial for recruitment of stem cells to many diseased organs. However, the therapeutic activity of SDF-1α is potentially limited by N-terminal cleavage at position-2 proline by a cell surface protein CD26/dipeptidyl peptidase-IV (DPP-IV). Parathyroid hormone (PTH) is a DPP-IV inhibitor and has been suggested as a promising agent for periodontal tissue repair. The purpose of this study was to explore the effects of a cell-free system comprising SDF-1α and scaffold plus PTH systemic application on periodontal tissue regeneration *in vivo*. The results showed that PTH/SDF-1α cotherapy improved the quantity of regenerated bone and resulted in better organization of ligament interface. We further investigated the possible mechanisms, and found that PTH/SDF-1α cotherapy enhanced CD90+CD34− stromal cells migration *in vivo*, increased the number of CXCR4 + cells in periodontal defects, induced early bone osteoclastogenesis and enhanced the expression of runt-related transcription factor 2 (Runx2), alkaline phosphatase (ALP) and collagen I (Col I) in newly formed bone tissue. In conclusion, this cell-free tissue engineering system with local administration of SDF-1α and systemic application of PTH could be employed to induce CD90+CD34− stromal cells recruitment and promote periodontal tissue regeneration.

Periodontitis is an infectious disease involving periodontal ligament and alveolar bone, which is characterized by gingival tissue inflammation and periodontal attachment loss, eventually leading to tooth loss^1^. In addition to the control of local inflammation within the periodontium and the progress of periodontitis, one major goal of periodontal therapy is to regenerate those lost tissues to their original form, architecture and function, which poses a challenging task to dentists^2^. A range of trails to regenerate the lost tissues have been attempted, with varying success in the clinical outcomes^3^,^4^,^5^. The major limitation appears to be the absence of adequate number of stem/progenitor cells to reconstruct periodontal ligament (PDL) and alveolar bone^3^. Cell delivery has been the classical approach in tissue regeneration. However, *ex vivo* manipulations of stem cells and cell seeding techniques have drawbacks for clinical applications such as high costs, time-consuming, risks for contaminations, pathogen transmission and tumorigenesis^6^,^7^,^8^,^9^. To overcome those limitations, recent studies have tried to mimic endogenous wound healing processes by activating the host’s own reparative capability and suggested the possibility of tissue regeneration by recruitment of endogenous stem cells to defected regions without transplantation of exogenous cells^10^,^11^. This strategy is called *in situ* tissue engineering which uses scaffolds alone or combined with bioactive factors to create a microenvironment that allows the body’s own cells to infiltrate and take over the scaffold and eventually integrate into native tissues^12^. For successful *in situ* tissue regeneration, an appropriate number of host stem cells must be recruited^13^. A wide set of chemokines have been applied *in vivo* to recruit endogenous mesenchymal stem cells (MSCs) for tissue regeneration. Among them, stromal cell-derived factor-1α (SDF-1α) has been recognized as the most important chemokine for the recruitment and homing of bone marrow-derived mesenchymal stem cells (BMSCs) throughout the mammalian system^14^.

SDF-1α, also known as CXCL12, plays an important role in recruitment of circulating stem cells into local injured tissues^15^. SDF-1α belongs to the C-X-C chemokine family, and exerts multiple biological functions through its receptor CXCR4, a G-protein coupled receptor^16^,^17^. SDF-1α/CXCR4 axis is important for embryonic organ development and essential for physiological functions, including blood homeostasis, inflammatory response, and bone remodeling^18^,^19^. SDF-1α has been shown to enhance the recruitment of intravenously infused extragenous stem cells into heart and brain ischemic tissues^20^,^21^. In the initial phase of bone repair, SDF-1α/CXCR4 axis mediates the recruitment of MSCs to the site of bone regeneration^15^. Our previous study demonstrated that local administration of SDF-1α could recruit stem cells to the periodontal defect, reduce inflammatory responses during the early phase of wound repair, and promote the quantity and quality of newly formed bone^22^. We also found that the injury itself could induce the production of endogenous SDF-1α. However, the production was limited and the concentration was relatively low. The extent and length of MSCs recruitment depends heavily on the duration and the concentration of SDF-1α release, thus application of exogenous SDF-1α may be a promising strategy to recruit circulating stem cells and precursor cells to periodontal defect and to promote tissue repair and regeneration.

SDF-1α is N-terminally cleaved at position-2 proline by a cell surface protein CD26/dipeptidyl peptidase-IV (DPP-IV)^23^. The truncated form of SDF-1α not only loses its chemotactic properties, but also blocks chemotaxis of full length SDF-1α^24^. DPP-IV is constitutively expressed on many hematopoietic cell populations and also found in a catalytically active soluble form in plasma^25^. Therefore, inhibition of DPP-IV is crucial to maintain the therapeutic activity of SDF-1α. Local application of SDF-1α in combination with DPP-IV inhibitor may be therapeutically beneficial for periodontal defect repair.

Recently, parathyroid hormone (PTH) has been shown to be a DPP-IV inhibitor and could enhance SDF-1α-driven homing of CXCR4+ stem cells to the ischemic heart^26^. PTH is the first anabolic drug approved by the US Food and Drug Administration for the treatment of osteoporosis. PTH was currently well recognized as an anabolic treatment option in the healing process of bony defects, besides its influence on stem cells migration and homing^27^,^28^. It has been well accepted that continuous exposure of PTH increases bone resorption, while intermittent administration of PTH stimulates new bone formation and improves microarchitecture of existing bone. A recent report demonstrated that intermittent PTH administration was able to reduce the number of inflammatory cells at the marginal gingival area and protect against periodontitis-associated bone loss in a rodent model^29^. Topical and intermittent administration of PTH recovered alveolar bone loss in rat experimental periodontitis and similar findings were obtained from a randomized clinical-trial in 40 patients with severe periodontitis^30^,^31^. These observations provide credence to the anabolic potential of intermittent application of PTH and its possible relation to reparative process occurring in the periodontium. Together with the potential of mobilizing large numbers of endogenous progenitor cells directly into the peripheral blood that will home to the site of injury and take part in tissue regeneration, PTH has been suggested as a promising agent for periodontal tissue repair^29^,^30^,^31^. We therefore hypothesized that intermittent PTH treatment combined with local application of SDF-1α may promote periodontal tissue regeneration.

Therefore, the purpose of this study was to explore the effects of a cell-free tissue engineering system comprising SDF-1α and scaffold plus PTH systemic application on the recruitment of CD90+CD34− stromal cells and periodontal tissue regeneration *in vivo*.

## Results

### Cooperative effects of PTH and SDF-1α on alveolar bone regeneration

**Figure 1:**
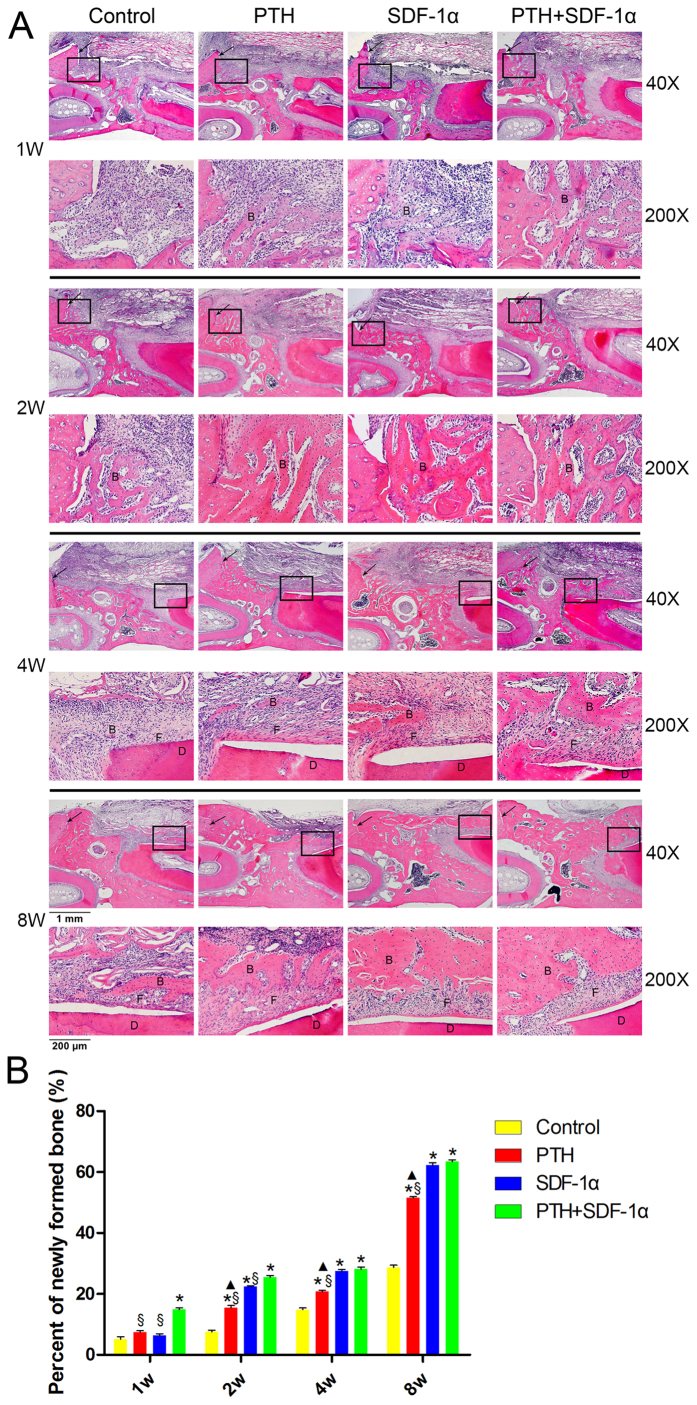
Promotion of alveolar bone formation by PTH and SDF-1α. (**A**) H&E staining of the periodontal defects at 1, 2, 4 and 8 weeks post-surgery. The visual fields framed by the black line were magnified in the images below. The black arrows represented the border of the defect. B indicated new bone. D displayed the dentine of the native root. F represented newly formed fibers. (**B**) Quantitative analysis of newly formed bone areas in the four groups at four time points. *P < 0.05 compared with control group; ^▲^*P* < 0.05 PTH group compared with SDF-1α group; ^§^*P* < 0.05 PTH + SDF-1α group compared with other experimental groups.

The effects of PTH and SDF-1α on alveolar bone formation were assessed by H&E staining and histomorphometric measurements (Fig. 1A,B). At 1 week post-surgery, no bone formed in control group, while a small amount of newly formed bone tissue could be observed on the edge of the defect in three experimental groups, and PTH + SDF-1α group formed more bones than the other three groups (*P* < 0.05). At week 2, more regenerated new bones formed in PTH + SDF-1α group than the other three groups (*P* < 0.05). The percentage of newly formed bone in both PTH and SDF-1α groups was significantly greater than that in control group (*P* < 0.05) and SDF-1α induced more bone formation than PTH (*P* < 0.05). At week 4, new bone formation at the edge of the defect could be observed in all four groups and a small amount of new bone could be observed near the root in control and PTH groups, while SDF-1α and PTH + SDF-1α groups formed more bones in the same location than the other two groups (*P* < 0.05), and there was no significant difference between SDF-1α and PTH + SDF-1α groups (*P* > 0.05). At week 8, remarkably more new bones formed in the whole defect in PTH + SDF-1α and SDF-1α groups than in PTH and control groups (*P* < 0.05) and there was no statistical difference between PTH + SDF-1α and SDF-1α groups (*P* > 0.05).

### Cooperative effects of PTH and SDF-1α on periodontal ligament and cementum regeneration

**Figure 2:**
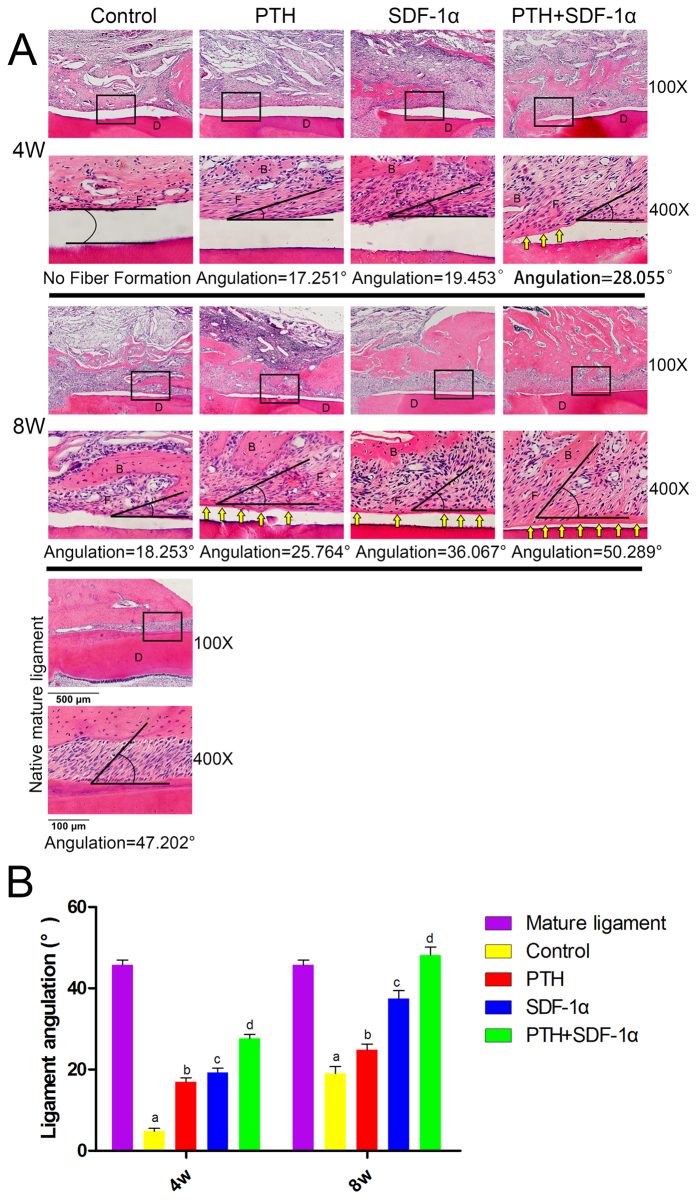
Promotion of fiber and cementum regeneration by PTH and SDF-1α. (**A**) H&E staining of the periodontal defects at 4 and 8 weeks post-surgery. The angle represented the angulation of regenerated ligament direction of each representative image. Yellow arrows indicated newly formed cementum. B indicated new bone. D displayed the dentine of the native root. F represented newly formed fibers. (**B**) Angulation analysis of regenerated ligament tissues. (**a–d**) *P* values of the angulation of native mature ligament compared with the fibers in four groups. 4 w: *P*^a^ < 0.001; *P*^b^ < 0.001; *P*^c^ < 0.001; *P*^d^ < 0.001; 8 w: *P*^a^ < 0.001; *P*^b^ < 0.001; *P*^c^ = 0.003; *P*^d^ = 0.314.

To quantitatively evaluate fiber formation and orientation of periodontal ligament, the angulation of fiber bundles against the root surfaces was measured and analyzed. Compared with the angulation of native mature ligament (mean = 47.2°)^32^, fibers in control, PTH and SDF-1α groups demonstrated significantly smaller angulation (week 4: *P*^a^ < 0.001; *P*^b^ < 0.001; *P*^c^ < 0.001, week 8: *P*^a^ < 0.001; *P*^b^ < 0.001; *P*^c^ = 0.003) (Fig. 2A,B). The fiber angulation in PTH + SDF-1α group at week 4 was significantly smaller than 47.2° (week 4: *P*^d^ < 0.001) whereas there was no statistical difference at week 8 in angulation between the native ligament tissue and the fibers in PTH + SDF-1α group (week 8: *P*^d^ = 0.314). These data demonstrated that SDF-1α combined with PTH could guide ligament orientation which is similar to the native mature ligaments. In addition to the fibers, newly formed cementum was observed. At week 4, no cementum was found in PTH, SDF-1α and control groups, while thin cementum could be found in PTH + SDF-1α group. At week 8, the newly formed cementum in PTH + SDF-1α became thicker, and thin cementum could be observed in both PTH and SDF-1α groups.

### PTH and SDF-1α promoted the engraftment of CXCR4+ cells *in vivo*

**Figure 3:**
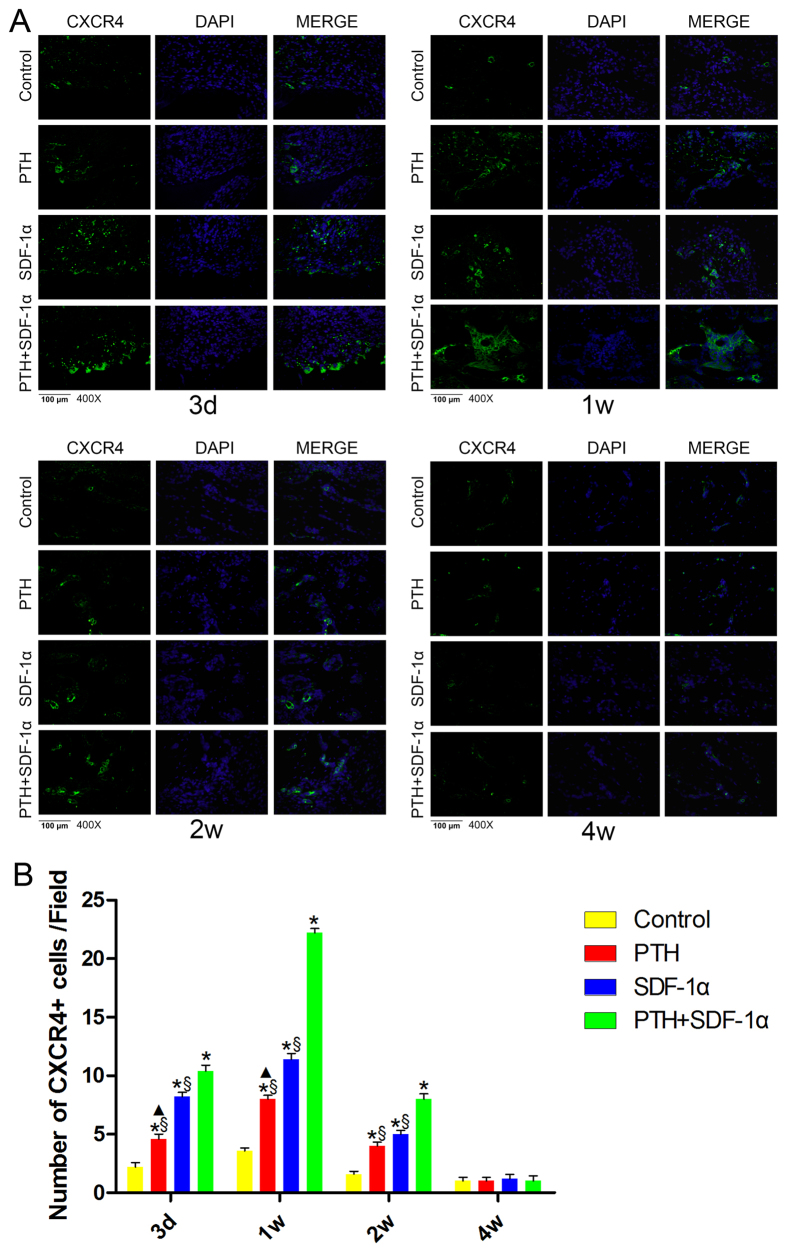
The combination of PTH and SDF-1α promoted the migration of CXCR4+ cells in periodontal defects. (**A**) Immunofluorescence staining of CXCR4+ cells in four groups at four time points. (**B**) Quantitative analysis of the number of CXCR4+ cells showed that PTH + SDF-1α promoted the recruitment of CXCR4+ cells than that in the other three groups at the early stage of healing process (day 3, week 1 and week 2). The number of CXCR4+ cells in SDF-1α group was larger than that in PTH group at day 3 and week 1, and there was no difference at week 2. At week 4, CXCR4+ cells were hardly detected. *P < 0.05 compared with control group; ^▲^*P* < 0.05 PTH group compared with SDF-1α group; ^§^*P* < 0.05 PTH + SDF-1α group compared with other experimental groups.

During the early stage of defect repair (day 3, week 1 and week 2 post-surgery), the number of CXCR4+ cells in control group was significantly smaller than that in the other three experimental groups (*P* < 0.05) (Fig. 3A,B). At day 3 and week 1, more CXCR4+ cells were observed in PTH + SDF-1α group compared with PTH or SDF-1α group (*P* < 0.05), and the number of CXCR4+ cells in SDF-1α group was significantly larger than that in PTH group (*P* < 0.05). Furthermore, the number of CXCR4+ cells in PTH + SDF-1α group peaked and was about 2-fold larger than that in SDF-1α group at week 1. CXCR4+ cells reduced obviously at week 2, while the number of CXCR4+ cells was still greater in PTH + SDF-1 group than that in PTH or SDF-1α groups (*P* < 0.05), and there was no significant difference between PTH and SDF-1α group (*P* > 0.05). Unsurprisingly, CXCR4+ cells were hardly detected at week 4 in all four groups. Moreover, in PTH + SDF-1α group at week 1, CXCR4+ cells were clustered and appeared forming some form of structures rather than distributed as individual cells. At week 4, the migrated CXCR4+ cells may form new structures which lost CXCR4 signaling as they matured.

### Cooperation of PTH and SDF-1α promoted the engraftment of CD90+CD34− stromal cells in periodontal defects

**Figure 4:**
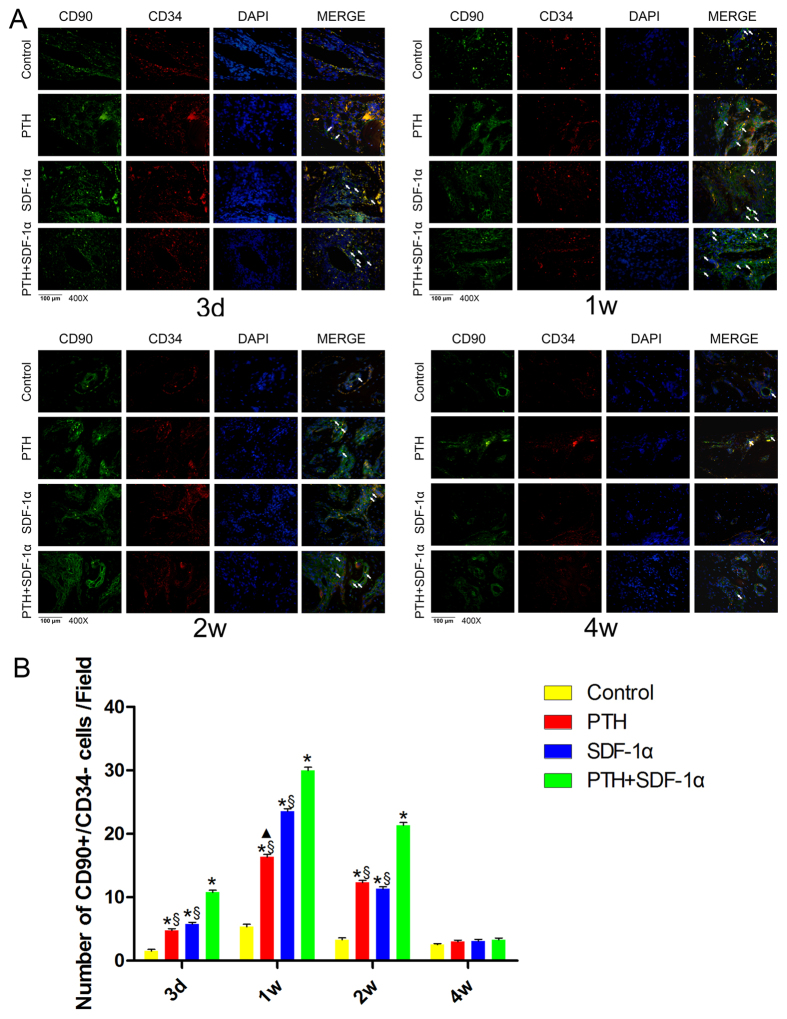
PTH and SDF-1α promoted the engraftment of CD90+CD34− stromal cells in periodontal defects. (**A**) Immunofluorescence double staining of the four groups at four time points. (**B**) Quantitative analysis of the number of CD90+CD34− stromal cells showed that SDF-1α combined with PTH increased the recruitment of CD90+CD34− stromal cells than the other three groups at day 3, week 1 and 2. The number of CD90+CD34− stromal cells peaked at week 1 and reduced at week 2. At week 4, MSCs were hardly detected. White arrow indicated CD90+CD34− stromal cells. *P < 0.05 compared with control group; ^▲^*P* < 0.05 PTH group compared with SDF-1α group; ^§^*P* < 0.05 PTH + SDF-1α group compared with other experimental groups.

We further characterized the response of host stromal cells to PTH and SDF-1α. Immunofluorescence staining for CD90 (green) and CD34 (red) was shown in Fig. 4A and Supplementary Fig. S1. The data showed that the number of CD90+CD34− stromal cells in control group was significantly smaller than that in other three experimental groups throughout the early healing process (*P* < 0.05) (Fig. 4B). At day 3, more CD90+CD34− stromal cells were detected in PTH + SDF-1α group compared with PTH or SDF-1α group (*P* < 0.05) while there was no significant difference between SDF-1α and PTH group (*P* > 0.05). The number of CD90+CD34− stromal cells peaked at week 1 and more CD90+CD34− stromal cells were found in PTH + SDF-1α group in comparison with PTH or SDF-1α group (*P* < 0.05). Moreover, the number of CD90+CD34− stromal cells in SDF-1 group was significantly greater than that in PTH group (*P* < 0.05). At week 2, though the number of CD90+CD34− stromal cells decreased obviously, PTH + SDF-1α group still recruited more CD90+CD34− stromal cells than PTH or SDF-1 alone (*P* < 0.05). Not surprisingly, CD90+CD34− stromal cells were hardly detected at week 4 in all four groups.

### Cooperative effects of PTH and SDF-1α on osteoclastogenesis

**Figure 5:**
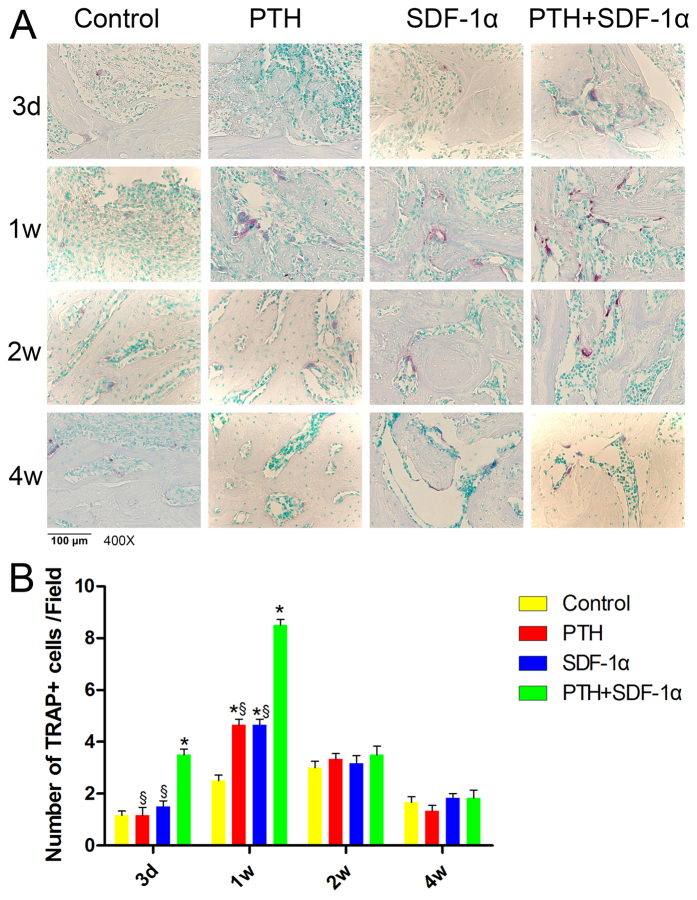
The effects of PTH and SDF-1α on osteoclastogenesis. (**A**) TRAP (red) staining at day 3, week 1, 2 and 4. (**B**) TRAP+ cells in four groups. *P < 0.05 compared with control group; ^▲^*P* < 0.05 PTH group compared with SDF-1α group; ^§^*P* < 0.05 PTH + SDF-1α compared with other experimental groups.

TRAP staining was used to evaluate osteoclast activity during wound healing. At day 3, PTH combined with SDF-1α significantly increased the number of TRAP+ cells in comparison with the other three groups (*P* < 0.05) (Fig. 5A, B). TRAP+ cells peaked at week 1, and the number of TRAP+ cells in PTH + SDF-1α, PTH and SDF-1α groups was significantly greater than that in control group (*P* < 0.05). Moreover, more TRAP+ cells was observed in PTH + SDF-1α group compared with PTH and SDF-1α groups (*P* < 0.05). At week 2, the number of TRAP+ cells decreased dramatically, and there was no significant difference among four groups (*P* > 0.05). Subsequently, TRAP+ cells were hardly detected at week 4 in all four groups.

### Cooperative effects of PTH and SDF-1α on Osteogenesis

**Figure 6:**
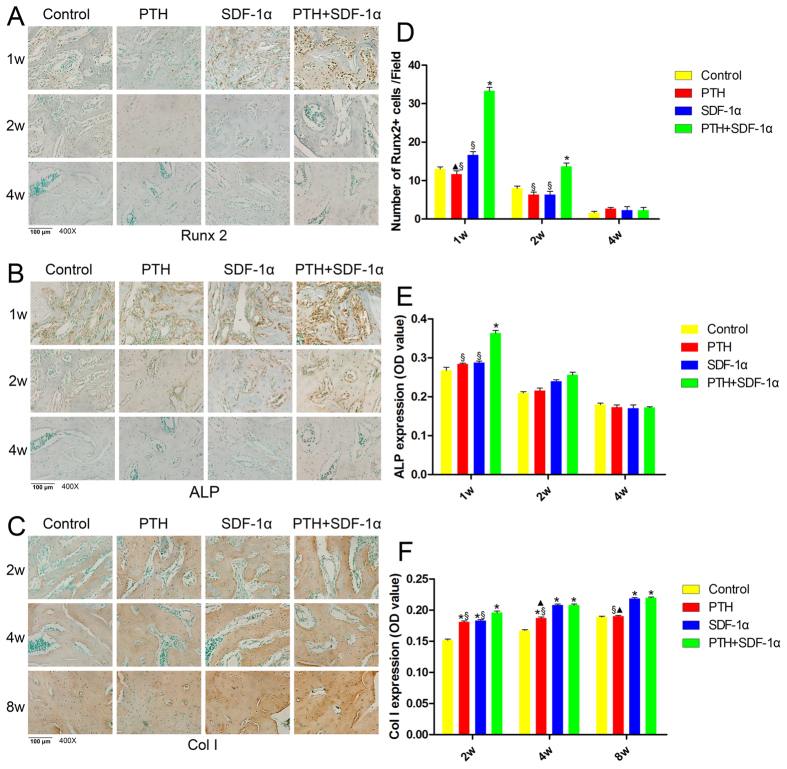
The effects of PTH and SDF-1α on osteogenesis. (**A,B**) Immunohistochemical staining of Runx2 (brown) and ALP (brown) at week 1, 2 and 4. (**C**) Immunohistochemical staining of Col I (brown) at week 2, 4 and 8. (**D–F**) Quantitative analyses of Runx2+ cells, ALP expression and Col I expression in four groups. *P < 0.05 compared with control group; ▲*P* < 0.05 PTH group compared with SDF-1α group; ^§^*P* < 0.05 PTH + SDF-1α compared with other experimental groups.

The effect of PTH and SDF-1α on osteogenesis was assessed by immunohistochemical staining. At week 1, more Runx2+ cells were observed in the periodontal defect area of PTH + SDF-1α group compared with the other three groups (*P* < 0.05), and SDF-1α promoted significantly more Runx2+ cells than PTH (*P* < 0.05) (Fig. 6A,D). At week 2, Runx2+ cells reduced obviously, while the number of Runx2+ cells in PTH + SDF-1α group was still higher than the other three groups (*P* < 0.05). At week 4, Runx2+ cells were hardly detected in all four groups. The expression of ALP was significantly enhanced in PTH + SDF-1α group in comparison with the other three groups at week 1 (*P* < 0.05) (Fig. 6B,E). At week 2, the level of ALP expression decreased obviously, and there was no significant difference among four groups (*P* > 0.05). At week 2, the osteogenic marker of Col I was highly expressed in PTH + SDF-1α, PTH and SDF-1α groups in comparison with control group (*P* < 0.05) (Fig. 6C,F), and the level in PTH + SDF-1α group was significantly higher than that in PTH and SDF-1α groups (*P* < 0.05). At week 4 and 8, SDF-1α and PTH + SDF-1α groups showed more Col I expression than the other two groups (*P* < 0.05), and there was no significant difference between SDF-1α and PTH + SDF-1α groups (*P* > 0.05). Negative controls for the IHC of Runx2, ALP and Col I were shown in Supplementary Figs S2–4.

## Discussion

In order to investigate the potential of endogenous stromal cell recruitment for periodontal tissue regeneration, an *in situ* tissue engineering system composed of collagen membrane scaffold loaded with SDF-1α and systemic application of PTH was developed in this study. The application of this cell-free tissue engineering system means that isolation and *ex vivo* manipulation of cells can be avoided, and periodontal tissue can be regenerated more efficiently and with less expense. To fully explore periodontal regeneration capability and the underlying mechanisms of this new cell-homing approach, we carried out a series of systematic experiments in this study. The results demonstrated that periodontal tissue regeneration was significantly promoted by collagen membrane loaded with SDF-1α and with systemic injection of PTH.

SDF-1α is an important chemokine that mediates injured tissue repair by stimulating progenitor cells recruitment^21^. Our previous studies suggested that local administration of SDF-1α may be a simple and safe technique for periodontal tissue regeneration^22^,^33^. However, a potential limitation for the therapeutic use of SDF-1α derives from its sensitivity to cleavage by DPP-IV^23^,^24^. Thus, stabilization of the active form of SDF-1α by the inhibition of DPP-IV may enhance the recruitment of stem cells/progenitor cells and tissue repair. Huber BC *et al*. reported that PTH significantly reduced the enzymatic activity of DPP-IV in a dose-dependent manner *in vitro*. PTH was also shown to lead to an increased cardiac SDF-1α protein level by inhibiting DPP-IV, thus enhancing stem cell migration into the ischaemic heart and improving cardiac function^26^. We investigated the *in vivo* periodontal regeneration capability of the cooperative treatment with PTH and SDF-1α in rat periodontal defects model.

The major goal of periodontal therapy is to regenerate periodontal tissues to their original form, architecture and function. In the present study, at week 8, remarkably more new bones formed in the whole defect in PTH + SDF-1α group than other three groups. Moreover, the combined application of PTH and SDF-1α could guide ligament orientation that is similar to the native mature ligaments and formed markedly more new cementum than PTH or SDF-1α alone. The regeneration of periodontal tissue is especially challenging, as it requires functionality of regenerated cementum-ligament-bone complexes around the periodontal defect. Our results indicated that SDF-1α combined with PTH have an excellent capacity to form bone, cementum, and functional periodontal ligament. Thus, our study demonstrated that combination of PTH and SDF-1α accelerated periodontal tissue regeneration in this cell free tissue-engineering system. To explore the mechanism of periodontal regeneration in the cell free tissue-engineering system, we further studied stromal cell migration, CXCR4 expression, early osteoclastogenesis and osteogenesis *in vivo*.

Known as Thy-1, CD90 is a cell-surface marker of stem and progenitor cells. Several studies have demonstrated that adult stem cells expressing CD90 possess high potential to undergo osteogenic differentiation^34^. CD34 is a glycosylated transmembrane protein and widely used as a marker in the identification and purification of human hematopoietic stem and progenitor cells. The CD90+CD34− cells were considered to be non-hematopoietic stromal cells, which may possess osteogenetic differentiation potential. Our results demonstrated that host-derived CD90+CD34− stromal cells were recruited and engrafted to defects by treatment with PTH plus SDF-1α. The combination of PTH and SDF-1α substantially increased the number of CD90+CD34−stromal cells compared with other groups. These results confirmed that PTH promoted the chemotactic capability of SDF-1α and enhanced stromal cells migration. The chemotactic properties of SDF-1α are mediated through interactions with its receptor CXCR4, which is widely expressed in many cells^33^. Besides MSCs and periodontal ligament stem cells (PDLSCs), CXCR4 has also been found in the membranes of inflammatory cells, osteoblasts, osteoclasts and their precursors^35^. Our results showed that PTH combined with SDF-1α substantially increased the number of CXCR4+ cells in the wound area. The binding of SDF-1α to CXCR4 eventually results in CXCR4+ cells moving towards higher concentration of SDF-1. The number of CXCR4+ cells significantly increased at day 3, peaked at week 1, and then slightly decreased after 2 weeks. These results indicate that complex interactions of SDF-1α with CXCR4 may exist in stromal cells recruitment and early stage of inflammatory cells response *in vivo.* Therefore, the cooperative effect of PTH and SDF-1α on CD90+CD34− stromal cells recruitment may be one explanation for the improved periodontal regeneration in our cell-free tissue engineering system.

The initiation and function of osteoclasts are critical for bone development and regeneration. Given that the activities of osteoclasts and osteoblasts are intertwined during normal bone remodeling, it is plausible that the anabolic action of PTH and SDF-1α is either directly or indirectly related to the osteoclasts^36^. Our results showed that the combination of PTH and SDF-1α promoted early, but not late osteoclastogenesis by inducing the expression of TRAP at day 3 and week 1, whereas TRAP+ cells dramatically reduced after 2 weeks. Bone apposition mostly occurs at sites that have recently gone resorption as these sites are able to attract multipotent osteogenic precursors that form osteoblasts. Additionally, the microenvironment at active resorption sites is rich in bone matrix proteins which are needed for the differentiation of precursor bone cells into osteoblasts. Thus, osteoclast generation, even if transient, may be necessary for PTH and SDF-1α induced anabolism in bone reconstruction and formation.

The process of osteogenic differentiation consists of three phases: proliferation with matrix formation, maturation and mineralization. During this process, orchestrated expression of osteogenic genes takes place. ALP, as an important marker for osteogenic differentiation, is expressed in the very early stages of osteogenic differentiation by stem cells or progenitor cells^22^,^37^,^38^. Runx2 is an osteoblast-specific transcription factor, which is implicated as a major regulator for osteoblast differentiation and osteogenic gene expression^39^. Our results demonstrated that ALP and Runx2 expression increased as early as day 7 when cells were treated with PTH and SDF-1α. This suggested that the combination of PTH and SDF-1α promoted osteoblast differentiation at an early stage. Col I is the most common collagen in human and plays an important role in bone formation and bone architecture^40^. In the present study, the relative gene expression level of Col I increased throughout the study interval for all groups, and PTH + SDF-1α group exhibited a higher expression of Col I compared with other groups. This observation indicates early deposition of bone matrix induced by PTH and SDF-1α. The immunohistochemical staining of bone-related markers in rat periodontal defects confirmed that the combination of PTH and SDF-1α enhanced bone formation.

After systemic application of PTH and local administration of SDF-1 in collagen membrane scaffolds to periodontal defects, we found that the cotherapy had the following synergistic effects: (1) induced the engraftment of host-derived CD90+CD34− stromal cells to participate in peirodontal repair process; (2) enhanced the number of CXCR4+ cells in periodontal wound area; (3) induced early bone osteoclastogenesis and enhanced the expression of osteogenic protein of Runx2, ALP and Col I in newly formed bone tissue; (4) improved the quantity of regenerated bone and resulted in better organization of ligament interface.

In conclusion, our results suggest that the combination of PTH and SDF-1α recruited more CD90+CD34−stromal cells and significantly promoted periodontal tissue regeneration *in vivo*. This strategy is expected to be a new method for clinical treatment of periodontal disease. In further studies, we plan to introduce a controlled-release scaffold to improve the performance of our cell-free tissue engineering system, and the exact mechanisms behind the synergistic effects of PTH and SDF-1α need to be further addressed on the basis of our current study.

## Methods

### Preparation of collagen membranes loaded with SDF-1α

Medical collagen repair membranes (Bote Biotechnology, Fujian, China) were cut into 5 × 4 × 1 mm pieces and placed in 96-well plates individually, injected with 100 μL SDF-1α (Santa Cruz Biotechnology, Santa Cruz, CA, USA) at a concentration of 50 μg/mL according to a previous study^37^ or PBS (control). The soaked collagen scaffolds were incubated at 4 °C overnight before grafting.

### Ethics statement

All animal experiments were conducted under the guide of the Care and Use of Laboratory Animals of the Chinese Science and Technology Ministry. This study was approved by the Committee on the Ethics of Animal Experiments of Shandong University (Permit Number: 201302070). All procedures were performed under pentobarbital sodium anesthesia.

### Rat periodontal fenestration defects model

**Figure 7:**
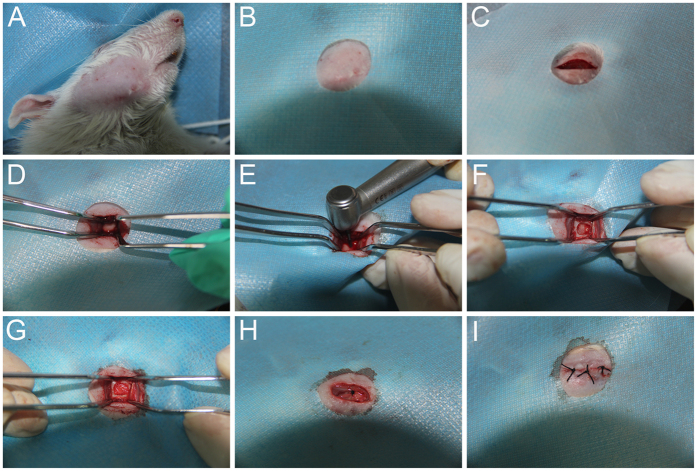
Overview of the surgical procedure. (**A**) Pre-operative skin preparation. (**B**) Disinfected the surgical site and covered with sterile drape. (**C**) A 2 cm long extra-oral incision was made parallel to the inferior margin of mandible. (**D**) The bone surface was exposed. (**E**) Preparation of the periodontal fenestration defect. (**F**) The created periodontal fenestration defect. (**G**) The collagen membrane placement in the periodontal fenestration defect. (**H**) The muscle was sutured. (**I**) The skin was sutured.

Sixty 8-week-old Wistar rats (220–250 g, male) were used in this study. In order to investigate the effect of the combination of PTH and SDF-1α on periodontal tissue regeneration, rats were numbered and divided randomly into two groups: intraperitoneal injected with human PTH (1–34) (Sigma-Aldrich, St Louis, MO, USA) at 40 μg/kg or vehicle (0.9% sodium chloride) on alternate days. Each rat has two defects at bilateral mandibles. Left-side defects were implanted with collagen membranes loaded with SDF-1α (Santa Cruz Biotechnology, Santa Cruz, CA, USA), while the right-side defects were implanted with collagen membranes loaded with PBS. Thus there were four groups: (1) control; (2) SDF-1α only; (3) PTH only; (4) PTH + SDF-1α, and there were 6 samples for each group at each time point. Surgical procedures were performed as previously described^22^. Briefly, the rats were anesthetized by intraperitoneal administration of pentobarbital sodium (40 mg/kg), and then a 2 cm long extra-oral incision was made parallel to the inferior margin of bilateral mandibles, and the subcutaneous tissues and masseter muscle were separated from bone surface. The buccal alveolar bone, periodontal ligament and cementum of the mandibular first molar roots were removed carefully using a dental round bur at low speed under physiological saline irrigation to prepare the periodontal fenestration defect (length ×  height  ×  depth: 5 mm  ×  4 mm  ×  1 mm). The wound defect was located in 1 mm behind the front of the mandible and 1 mm below the crest of the alveolar bone. Attention was given to avoid breaking the roots. After sufficiently cleaning the wound, the collagen membranes loaded with SDF-1α or PBS were implanted into the defects, and then the muscle and skin were sutured respectively (Fig. 7). After surgery, injections of PTH (1–34) (40 μg/kg) intermittently or vehicle were administered to each rat before sacrifice.

### Sample harvesting and preparation for histological analyses

Rats were sacrificed at 3 days, 1 week, 2, 4 and 8 weeks after surgery. There were 12 rats at each time point. Animals were anesthetized with pentobarbital sodium (40 mg/kg) and perfused through the heart with 100 ml saline and then 250 ml of 4% paraformaldehyde in phosphate buffer (pH 7.4). The bilateral mandibles of each rat were excised carefully and fixed with 4% paraformaldehyde in phosphate buffer at 4 °C for 1 day, and then decalcified for 4 weeks in 10% ethylenediamine tetraacetate (EDTA) at 4 °C. After completion of decalcification, the tissues were dehydrated using a gradient ethanol solution and immersed in paraffin for 2 h. After embedding, the tissues were cut in a coronal direction to make 4 μm-thick serial sections in the buccal-lingual plane. All the sections were prepared for staining and analysis.

### Histological analysis

To determine periodontal tissue regeneration of each group, every tenth section was stained with hematoxylin and eosin (H&E; Solarbio, Beijing Solarbio Science & Technology, Beijing, China) and the remaining slices were used for immunohistochemistry and immunofluorescence. Specimens were observed under a BX53 microscope (Olympus, Tokyo, Japan) and measured with Image pro-plus 6.0 Software (Media Cybernetics, Silver Spring, MD, USA). The newly formed bone area measurement was normalized by the percentage of area of newly formed bone/area of defect × 100%. The newly formed functional fibers were measured by the angular orientation of fibrous ligament tissues.

### Immunofluorescence staining

Immunofluorescence staining of CXCR4 and double immunofluorescence staining of CD90/CD34 was employed to identify CXCR4+ cells and CD90+CD34− stromal cells. For immunofluorescence staining, rabbit polyclonal anti-CXCR4 antibody (1:100 dilution, ab7199; Abcam, Cambridge, MA, USA), rabbit monoclonal anti-CD34 antibody (1:200 dilution, ab81289; Abcam) and mouse monoclonal anti-CD90 antibody (1:400 dilution, ab225; Abcam) were used as primary antibodies. Alexa Flour 488-conjugated Affinipure Goat Anti-Rabbit IgG (H + L) (Proteintech, Chicago, IL, USA), Alexa Flour 488-conjugated Affinipure Goat Anti-Mouse IgG (H + L) (Proteintech) and Alexa Flour 594-conjugated Goat Anti-Rabbit IgG (H + L) (Proteintech) were used as secondary antibodies. 4′,6-diamidino-2-phenylindole (DAPI) (Solarbio) was used to stain cell nuclei.

### Immunohistochemical staining

Osteogenesis was evaluated by immunohistochemical analysis for ALP, Runx2 and Col I. For immunohistochemical staining, rabbit polyclonal anti-Runx2 antibody (1:200 dilution, ab23981; Abcam), rabbit polyclonal anti-ALP antibody (1:100 dilution, ab95462; Abcam) and rabbit polyclonal anti-Col I antibody (1:200 dilution, ab34710; Abcam) were used as the primary antibodies. Goat anti-rabbit IgG H&L (horseradish peroxidase) (1:100 dilution, ab6721; Abcam) was used as the secondary antibody. Immunoreactions were detected with DAB (Solarbio). Cell nuclei were stained with methyl green.

### TRAP staining

The number of tartrate-resistant acid phosphatase (TRAP) positive cells was detected to examine osteoclastogenesis. For TRAP staining, a leukocyte acid phosphatase kit (Sigma-Aldrich) was used according to the manufacturer’s instructions, and cell nuclei were stained with methyl green.

After staining, sections were imaged under the BX53 microscope. Three sections in different positions (anterior margin, central, posterior margin) of every sample were selected, and three randomly selected non-overlapping microscopic fields from each section were picked out. The number of CD90+CD34− stromal cells, CXCR4-positive cells, TRAP-positive cells and Runx2-positive cells were counted and the mean optical density (OD) of ALP and Col I staining was measured by Image pro-plus 6.0 software.

### Statistical analysis

Data were expressed as the mean ± standard error of the mean and were analyzed using SPSS software (SPSS Inc., IL, USA). One-way ANOVA was used to determine the significant differences in the data among the four groups, followed by a Bonferroni multiple comparison test for pairwise comparison. Statistical probability of *P* < 0.05 was considered significant.

## Additional Information

**How to cite this article**: Wang, F. *et al*. PTH/SDF-1α cotherapy induces CD90+CD34− stromal cells migration and promotes tissue regeneration in a rat periodontal defect model. *Sci. Rep.* **6**, 30403; doi: 10.1038/srep30403 (2016).

## Supplementary Material

Supplementary Information
