# Supplementary material for: PTH/SDF-1α cotherapy induces CD90+CD34− stromal cells migration and promotes tissue regeneration in a rat periodontal defect model
Source: Sci Rep. 2016 Aug 2;6:30403. doi: 10.1038/srep30403 (PMC4969616; doi:10.1038/srep30403)
Supplement: Supplementary Information [file srep30403-s1.pdf]

PTH/SDF-1α cotherapy induces CD90+CD34- stromal cells migration and promotes tissue regeneration in a rat periodontal defect model

Fang Wang[1,2,a], Lingqian Du[1,2,a], Shaohua Ge[1,2*]

[1]Shandong Provincial Key Laboratory of Oral Tissue Regeneration, School of Stomatology, Shandong University, Jinan, China

[2]Department of Periodontology, School of Stomatology, Shandong University, Jinan, China

[*] Correspondence: Shaohua Ge

Shandong Provincial Key Laboratory of Oral Tissue Regeneration, Department of Periodontology, School of Stomatology, Shandong University, 44-1 Wenhua Road West, Jinan 250012, China

Tel +86 531 8838 2123

Fax +86 531 8838 2923

Email shaohuage@sdu.edu.cn

Running header: Effects of PTH and SDF-1α on periodontal regeneration

[a] These authors contributed equally.

Supplementary Figures

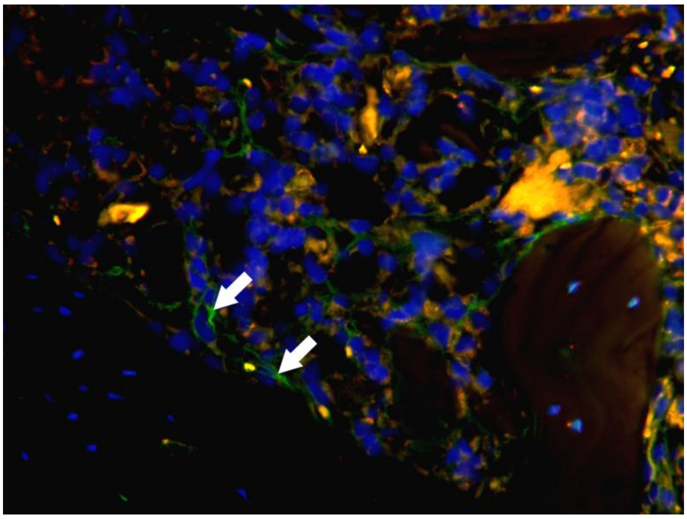

Figure S1 Immunofluorescence double staining of CD90+CD34- stromal cells

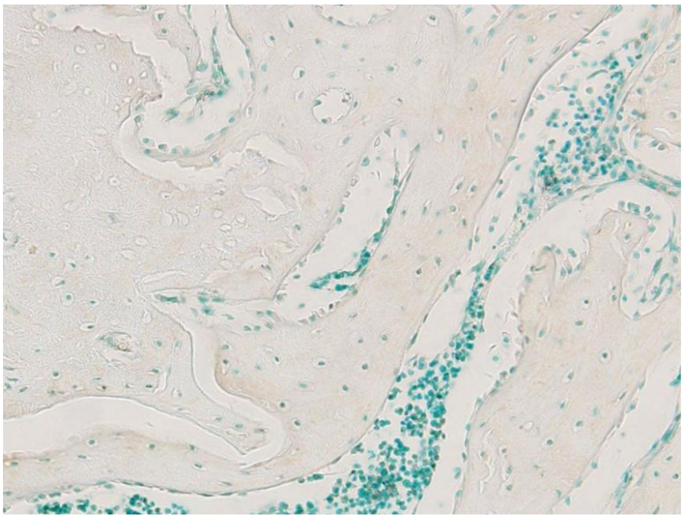

Figure S2 Negative control for the IHC of Runx2

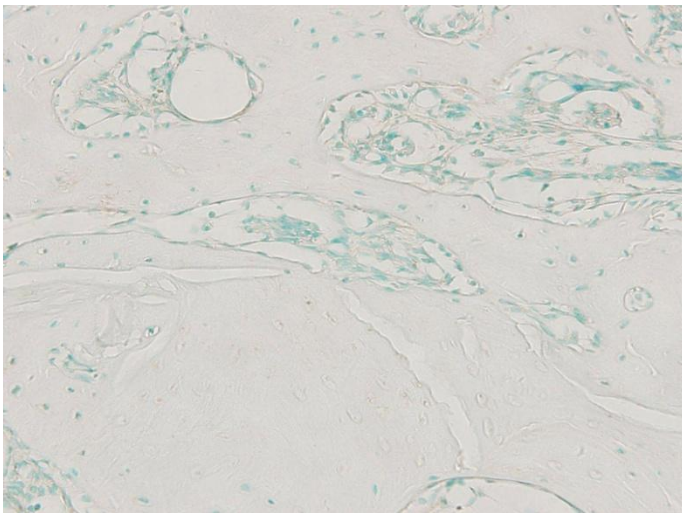

Figure S3 Negative control for the IHC of ALP

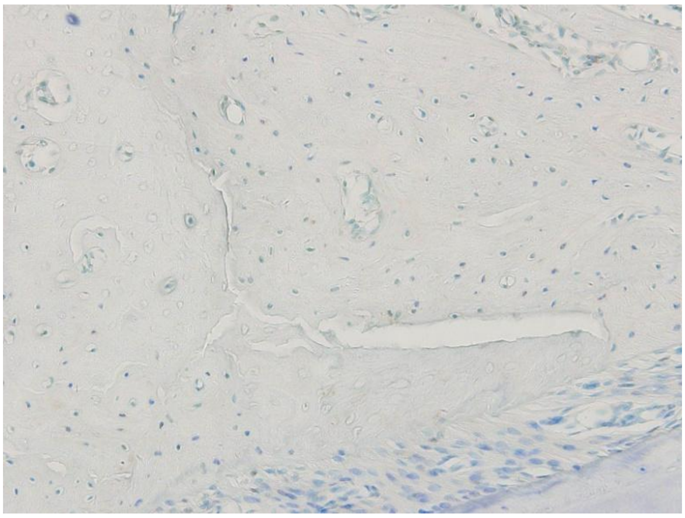

Figure S4 Negative control for the IHC of Col I
